# Quercetin in metabolic diseases: mechanisms, therapeutics, and multidimensional frontiers

**DOI:** 10.3389/fendo.2026.1800322

**Published:** 2026-04-13

**Authors:** Qingliang Song, Lianghao Liu, Qinhe Yang, Maoxing Pan, Yupei Zhang

**Affiliations:** 1 School of Traditional Chinese Medicine, Jinan University, Guangzhou, China; 2 The First Affiliated Hospital of Jinan University, Guangzhou, China

**Keywords:** metabolic disorders, molecular mechanisms, quercetin, recent progress, therapeutic applications

## Abstract

**Background:**

Metabolic diseases represent a significant global public health concern, imposing substantial burdens on healthcare systems, economies, and patient quality of life. Current treatments have limitations, underscoring the need for safer alternatives. Quercetin, a natural flavonoid with favorable human tolerability, shows promise for metabolic disorder management.

**Purpose:**

This review critically evaluates the existing evidence on quercetin’s role in metabolic disease management, summarizing its pharmacological advancements and clinical data in treating nine metabolic disorders: diabetes mellitus (DM), metabolic dysfunction-associated fatty liver disease (MAFLD), obesity, atherosclerosis, hyperuricemia, gouty arthritis, hyperlipidemia, osteoporosis, and polycystic ovary syndrome (PCOS).

**Methods:**

We systematically reviewed studies (2003-2025) from Web of Science, PubMed, Science Direct, and CNKI reporting quercetin’s effects in metabolic diseases.

**Results:**

Quercetin exhibits multifaceted pharmacological activities, including anti-inflammatory, antioxidant, antiapoptotic, hypolipidemic, and hypoglycemic effects. This underpins its therapeutic potential against nine metabolic disorders. Furthermore, emerging nanodelivery systems have demonstrated enhanced bioavailability, stability, and overall efficacy of quercetin while mitigating its dose-dependent toxicity.

**Conclusion:**

Quercetin shows considerable promise in the intervention of metabolic diseases. However, current research lacks mechanistic depth, bioavailability enhancement data, and clinical validation Additionally, clinical studies validating its therapeutic efficacy remain scarce. Further mechanistic investigations and randomized controlled trials are imperative to elucidate quercetin’s precise mechanisms and substantiate its clinical potential in metabolic disease management.

## 1 Introduction

Disruption of normal metabolic processes in the human body leads to dysregulation in the metabolism of proteins, fats, and carbohydrates, resulting in metabolic disorders (1). Urbanization, unhealthy diets, and sedentary lifestyles have driven rising prevalence of metabolic diseases, such as diabetes mellitus (DM), metabolic dysfunction-associated fatty liver disease (MAFLD), atherosclerosis (AS), obesity, hyperlipidemia, hyperuricemia (HUA), and osteoporosis (OP), has been rising steadily, and currently affect nearly 2 billion people worldwide (2, 3).

These disorders share common pathogenic features: glucose/lipid dysregulation, insulin resistance (IR), and organ damage. These challenges drive ongoing research to identify novel therapeutic targets for developing safer and more effective treatments. Although existing drugs—such as triglyceride-lowering agents, urate-lowering therapies, hypoglycemic medications, and insulin sensitizers—can mitigate metabolic abnormalities, there remains an urgent need for interventions that reduce the prevalence, complications, and mortality associated with these conditions (4, 5).

Plant-derived compounds are increasingly attractive due to multi-target effects and favorable safety profiles. Among these, flavonoids exhibit pleiotropic bioactivities, with quercetin being the most extensively studied (6, 7).

This review summarizes quercetin’s characteristics, bioavailability, mechanisms, and clinical evidence in nine metabolic diseases, including DM, MAFLD, obesity, AS, HUA, gouty arthritis, hyperlipidemia, OP, and PCOS. The aim is to offer valuable insights for future research and development of quercetin-based therapeutics.

## 2 Materials and methods

**Figure 1:**
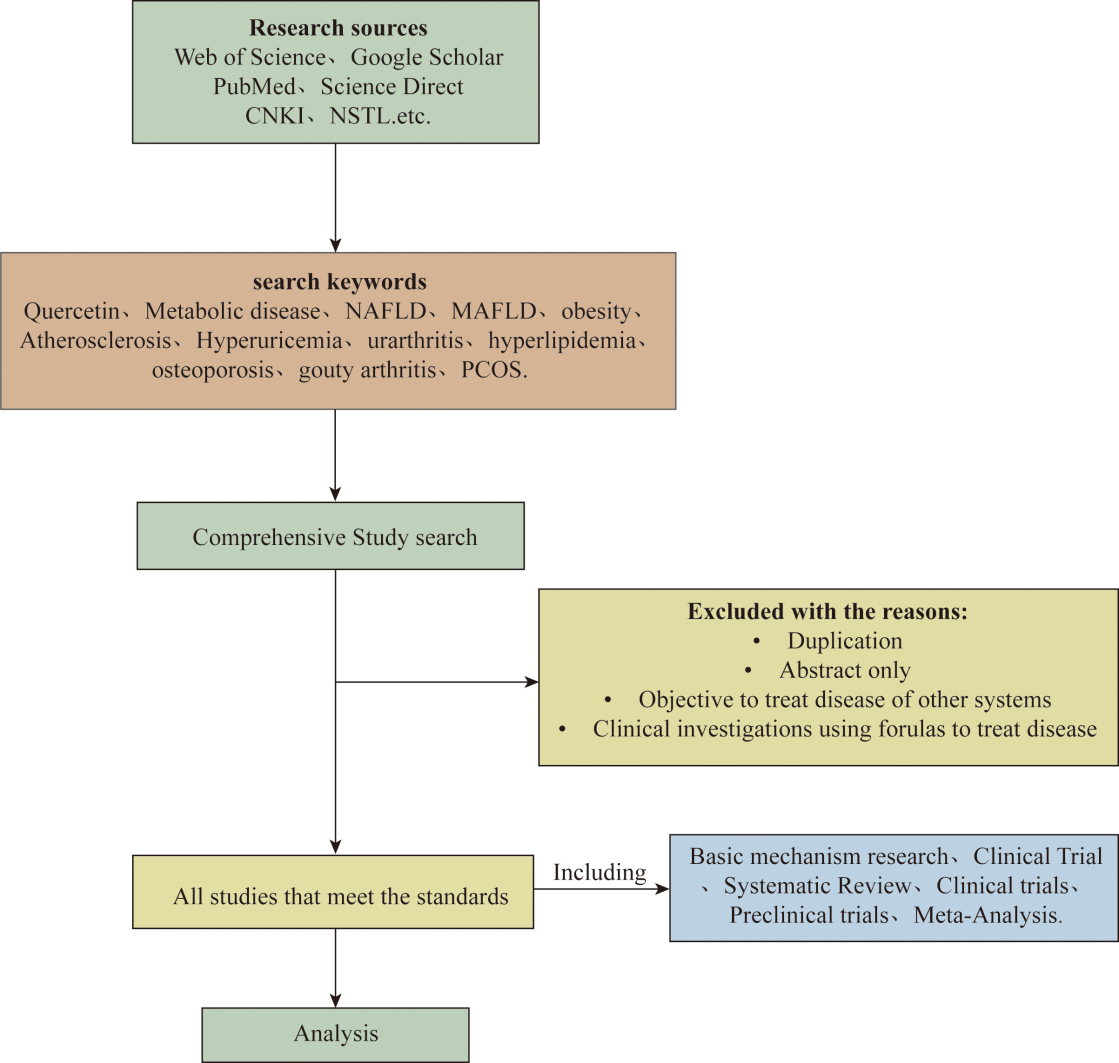
Flow chart detailing the selection of this study.

We conducted systematic literature searches (2003-2025) across Web of Science, PubMed, Science Direct, and CNKI using keywords: ‘quercetin’ combined with metabolic disease terms. Inclusion criteria: animal studies, cellular research, and clinical trials. Exclusion: editorials, conference abstracts, and incomplete data. the flowchart of the screening process is shown in **Figure 1**.

## 3 Source and fundamental characteristics of quercetin

Quercetin (3,3’,4’,5,7-pentahydroxyflavone) is a naturally occurring flavonol compound that exhibits Quercetin (C_15_H_10_O_7_, MW 302.24) is a yellow flavonol with limited water solubility (60 mg/L) but good organic solvent solubility.but shows good solubility in various organic solvents, including ethanol, methanol, and diethyl ether (8). The molecular structure contains characteristic phenolic hydroxyl groups arranged in both meta- and ortho-positions on the benzene rings, along with a conjugated double bond system and a carbonyl group, which collectively contribute to its significant biological activity and antioxidant properties (9).

**Figure 2:**
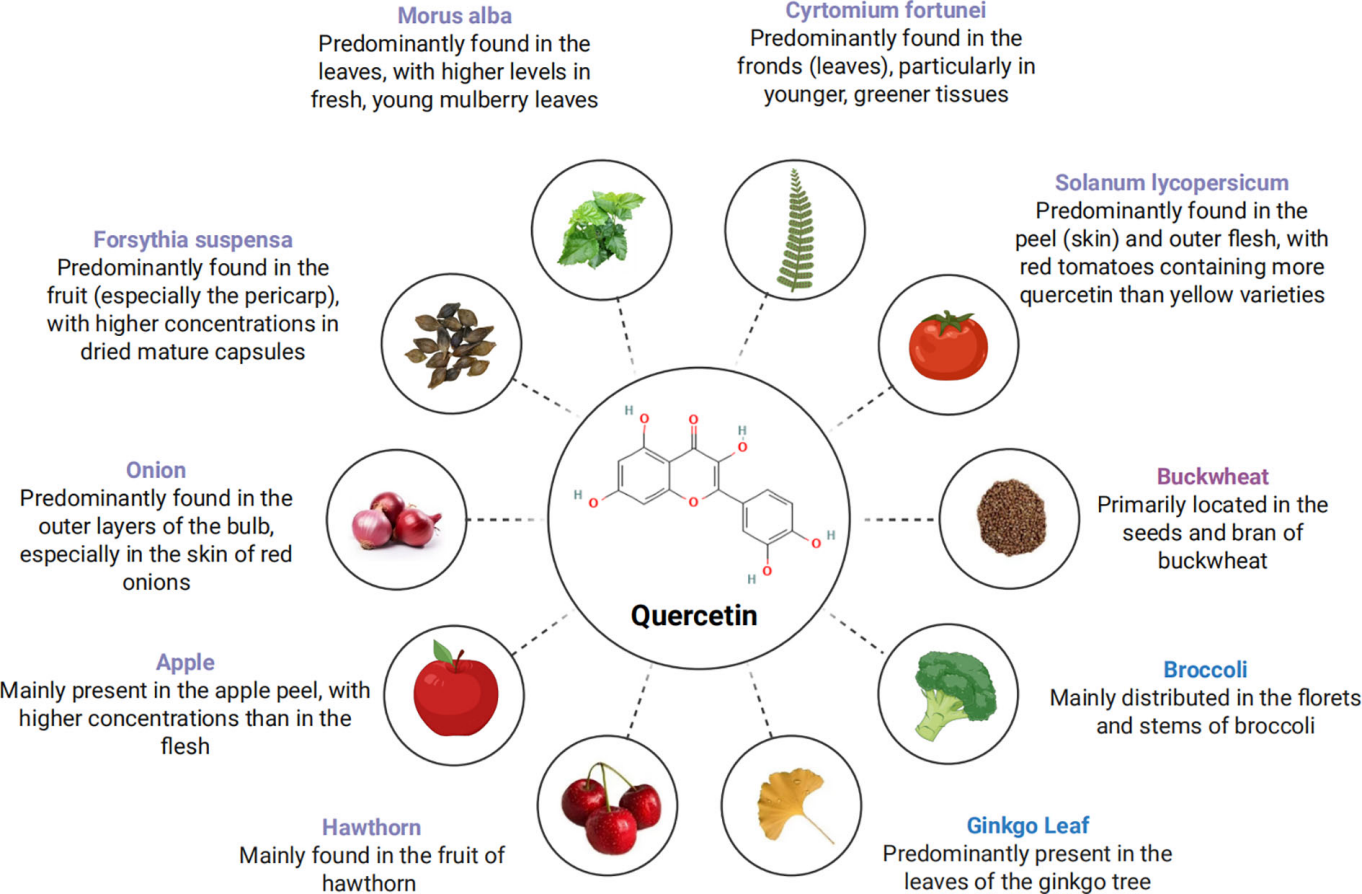
The main source of quercetin.

**Figure 3:**
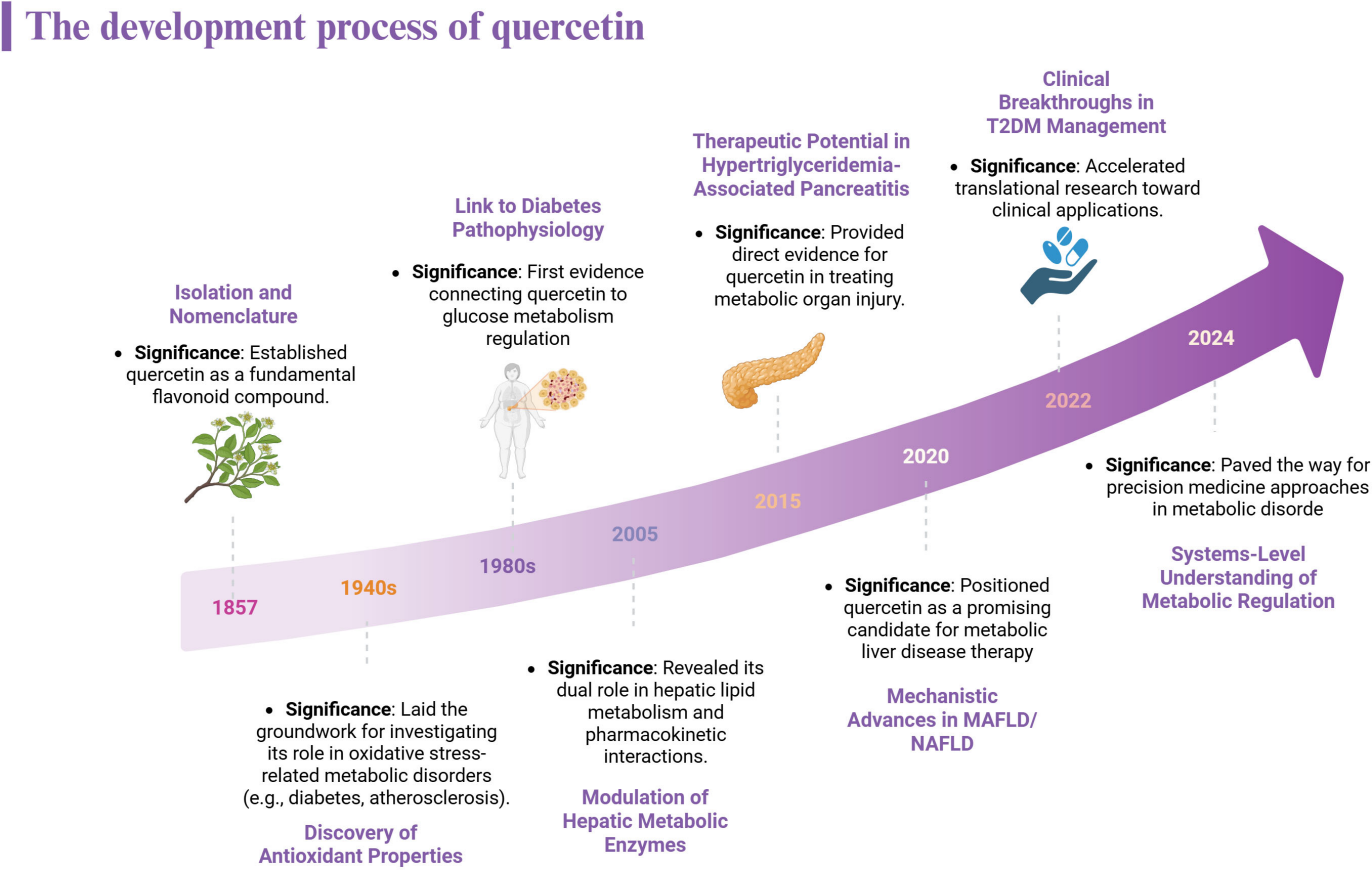
The development process of quercetin.

In natural sources, quercetin predominantly exists in glycosylated forms, where sugar moieties are attached to enhance its hydrophilicity and stability. This flavonoid is widely distributed throughout the plant kingdom and is commonly found in many dietary components. Notable food sources Dietary sources include onions, apples, berries, tea, and red wine (**Figure 2**) (10–12). The compound also occurs in numerous medicinal plants used in traditional medicine systems, with significant quantities present in Forsythia suspensa, Ginkgo biloba leaves, and Morus alba leaves, among others (13) Dietary intake of quercetin typically accounts for a substantial portion (60-75%) of total flavonol consumption in most populations, though exact levels vary depending on dietary patterns and regional food preferences (14). The development process of quercetin is shown in **Figure 3**.

**Figure 4:**
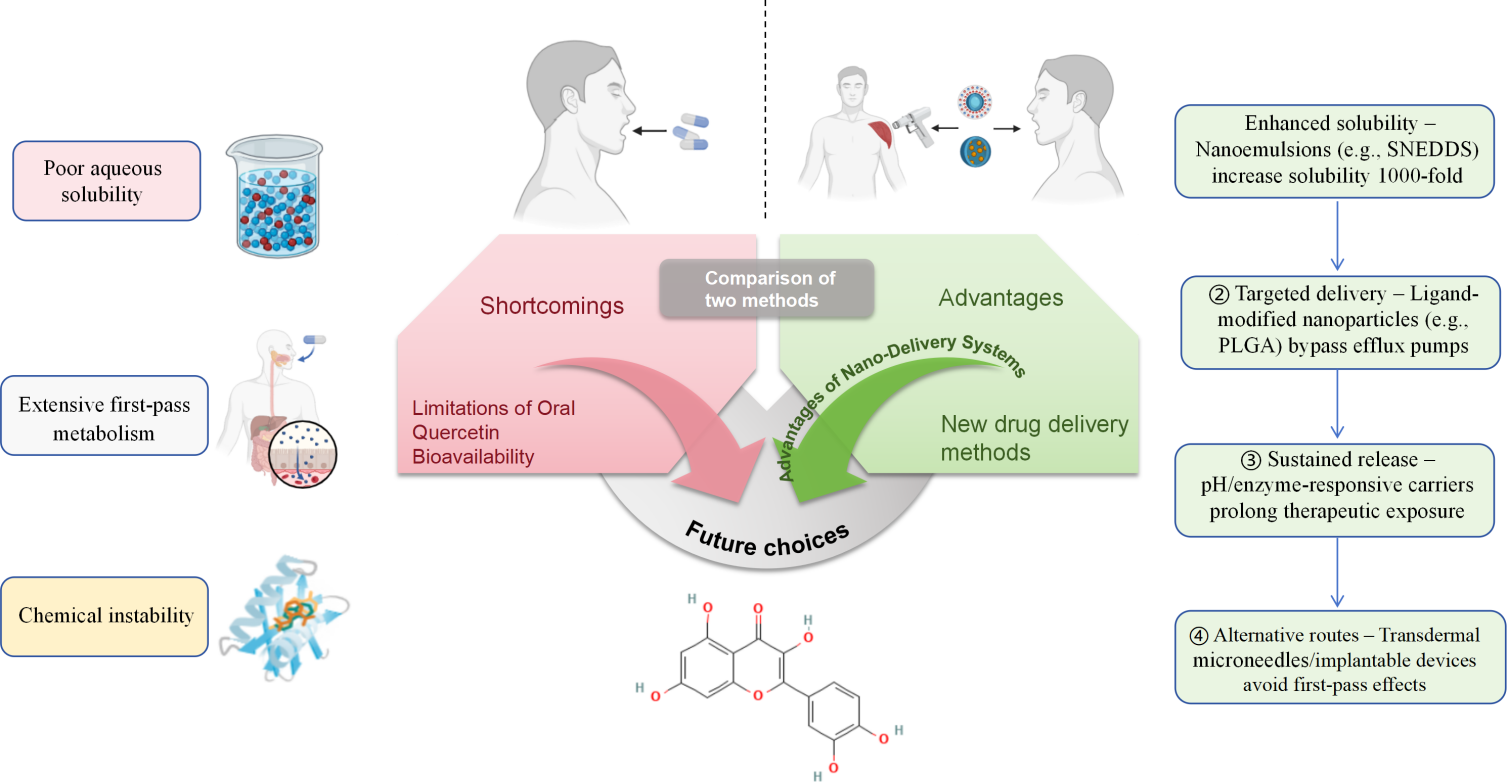
Comparison of oral and novel drug delivery methods.

The extraction of quercetin from natural sources can be achieved through various methodologies. Extraction methods range from traditional alkaline/acid precipitation to modern techniques (microwave, ultrasound, supercritical fluid extraction). These advanced methods generally offer improved efficiency and reduced processing times compared to conventional extraction approaches (15). The stability of quercetin is known to be influenced by multiple environmental factors, Quercetin stability is compromised by alkaline pH, high temperature, and metal ion chelation; encapsulation improves stability (**Figure 4**), particularly using essential oil-based delivery systems, can significantly improve quercetin stability under challenging conditions (16, 17).

## 4 Metabolism and bioavailability of quercetin

Oral quercetin undergoes extensive phase II metabolism (glucuronidation, sulfation, methylation) in the small intestine and liver, with microbial biotransformation in the colon. Metabolites peak at 0.6-0.8 h, with terminal half-life ~3.5 h (18, 19). The absorbed quercetin and its metabolites are then partially transported via the hepatic portal vein to the liver for further biotransformation. Hepatic phase I metabolism, mediated by cytochrome P450 enzymes, involves oxidation, reduction, hydrolysis, and hydration reactions (20). Phase II metabolism predominantly consists of conjugation reactions (glucuronidation, sulfation, and methylation) that enhance hydrophilicity and reduce potential toxicity. Following hepatic metabolism, quercetin and its metabolites are transported into systemic circulation and bile via multidrug resistance-associated proteins (MRPs) (21). Biliary metabolites may undergo enterohepatic recirculation through the common bile duct or be excreted in feces. In the large intestine, unabsorbed quercetin from both intestinal and hepatic sources undergoes microbial biotransformation, including demethylation, hydroxylation, deglycosylation, decarboxylation, and ring cleavage, yielding low molecular weight phenolic compounds with improved absorption characteristics (22). Ultimately, quercetin is excreted primarily as glucuronide or sulfate conjugates through expired CO_2_, feces, and urine (23).

Quercetin exhibits rapid metabolism and a short plasma half-life. Metabolites become detectable in plasma within 30 minutes post-ingestion, with substantial elimination occurring within 24 hours (10). The predominant plasma metabolites are quercetin-3’-sulfate and quercetin-3-glucuronide, reaching peak concentrations at 0.8 and 0.6 hours, respectively (10). Human pharmacokinetic studies demonstrate high oral clearance (3.5×10^4^ L/h) with a mean terminal half-life of 3.5 hours (24). Acute consumption of quercetin-rich foods yields total plasma concentrations of 72–193 nmol/L, while prolonged supplementation (1,095 mg/day for 3 days) achieves levels up to 1,430 nmol/L (25–27).

Food matrix significantly influences quercetin bioavailability. Comparative studies show food matrix significantly affects bioavailability (e.g., grape juice > wine > vegetable homogenate)., respectively (10 mg/70 kg body weight) (28). Considerable interindividual variability in bioavailability exists, attributable to endogenous factors (sex, age, intestinal permeability) and exogenous factors (food matrix, dietary fat content, glycosylation patterns).

## 5 A novel delivery method for quercetin

Despite being one of the most extensively studied flavonoids with promising therapeutic potential against metabolic disorders, quercetin’s clinical application is significantly limited by its poor stability, low water solubility, limited oral bioavailability, and rapid metabolism. Enhancing its absorption and metabolic activity represents an important direction for future research. Currently, most studies on quercetin’s anti-metabolic disease effects remain at the preclinical stage using animal models and *in vitro* systems, with limited clinical evidence available. Notably, formulation with sunflower lecithin in food-grade delivery systems enhances oral absorption by approximately 20-fold, demonstrating the potential of nanotechnological approaches to improve therapeutic utilization (29).

Notably, biocompatible and biodegradable nanoparticles have emerged as effective delivery systems for phytochemicals. Encapsulating quercetin in various nanocarriers (biopolymers, nanoemulsions, liposomes, micelles) can significantly improve its bioavailability and bioactivity while reducing dosing frequency (30–33). These nano-delivery systems mitigate quercetin’s degradation during processing and storage, and enable sustained release during digestion, effectively addressing its stability and bioavailability limitations (34, 35). In T2DM rat models, quercetin nanoemulsion (Que-NE) demonstrated superior release profiles and oral bioavailability compared to free quercetin, exhibiting remarkable protective and therapeutic effects on body weight management, glycemic control, lipid profile improvement, and pancreatic β-cell protection. Similarly, nanoencapsulated quercetin showed enhanced anti-obesity efficacy by overcoming the limitations of plain quercetin. However, current research on nanocarrier-delivered quercetin remains scarce, particularly for metabolic diseases other than T2DM and obesity. Importantly, safety concerns represent a major challenge for medical applications of nanoparticles, as each nanocarrier possesses unique characteristics, loading capacities, and potential drawbacks. The selection of appropriate nanocarriers requires careful consideration of multiple factors, including payload compatibility, optimal dosage, administration route, and frequency, as well as potential side effects and toxicity. Therefore, comprehensive investigations into the stability, safety, and biological interactions of nanoformulated quercetin are imperative.

To address the long-standing limitation of poor bioavailability, the development of next-generation intelligent nanodelivery systems has emerged as a major frontier in quercetin research. Compared with conventional nanocarriers, stimuli-responsive platforms enable spatiotemporally controlled drug release by exploiting the pathological microenvironment characteristic of metabolic diseases. In the acidic inflammatory milieu associated with diabetes and MAFLD, pH-responsive nanocarriers—such as carboxylated mesoporous silica and polyhistidine-modified liposomes—can selectively release quercetin at lesion sites with pH values of 6.5–6.8 while remaining stable under physiological conditions (pH 7.4), thereby enhancing local drug accumulation and minimizing systemic exposure (36). In atherosclerotic plaques characterized by elevated reactive oxygen species (ROS), ROS-responsive nanocarriers incorporating thioether or selenoether linkages enable oxidative stress–triggered cascade release, facilitating the targeted delivery of quercetin to the plaque core (36). More advanced strategies include glucose-responsive delivery systems based on phenylboronic acid–diol dynamic covalent interactions, which accelerate quercetin release under hyperglycemic conditions and thereby establish a closed-loop regulatory paradigm in which higher glucose levels trigger faster drug release—an approach with significant translational potential for glycemic fluctuation management in diabetes. In addition, biomimetic nanocarriers derived from exosomes or cell membrane coatings provide innovative platforms for cell-specific quercetin delivery due to their superior biocompatibility, low immunogenicity, and intrinsic tissue-targeting capabilities (37). Notably, macrophage membrane–coated nanoparticles can actively home to atherosclerotic plaques and fatty liver tissues, enabling precise therapeutic intervention.

Beyond improving delivery efficiency, another emerging frontier of quercetin nanotechnology lies in its application as a multimodal therapeutic platform. By integrating quercetin with complementary intervention strategies, these systems can achieve synergistic therapeutic outcomes and broaden the scope of potential indications. At the interface of tumor metabolic reprogramming and obesity, photothermal–chemotherapeutic nanoplatforms co-loading near-infrared–responsive photothermal agents (e.g., gold nanorods or black phosphorus quantum dots) with quercetin exploit localized photothermal effects to promote white adipose tissue browning while simultaneously enhancing the lipolytic regulatory activity of quercetin, thereby providing a noninvasive physico-pharmacological strategy for obesity treatment. Considering the chronic and progressive nature of metabolic diseases, long-acting sustained-release implantable systems—such as poly(lactic-co-glycolic acid) (PLGA) microspheres or *in situ* forming hydrogels—allow continuous quercetin release for weeks to months, addressing adherence challenges associated with frequent dosing. Injectable thermosensitive hydrogels, for instance, have demonstrated the ability to achieve single-administration, long-term controlled release in diabetic wound-healing models (38). A particularly forward-looking direction involves the construction of theranostic nanoplatforms that integrate quercetin delivery with real-time disease monitoring. Nanocarriers labeled with near-infrared fluorescent quantum dots or magnetic resonance imaging contrast agents enable dynamic tracking of drug distribution and accumulation within target tissues while simultaneously providing feedback on therapeutic efficacy, thereby supporting precision medicine and individualized dosing strategies for metabolic diseases (39). The systematic integration of these multidimensional technologies is expected to drive the strategic transformation of quercetin from a traditional phytochemical into an intelligent nanomedicine.

## 6 Mechanisms of quercetin in treating metabolic diseases

Detailed preclinical evidence regarding disease models, signaling pathways, and therapeutic indices is provided in **Table 1**.

**Table 1:** Basic research evidence of quercetin in metabolic and chronic diseases.

Type of disease	Quercetin formulation	Experimental model	Molecular targets/mechanisms	Outcomes	References
Diabetes Mellitus (T2DM)	Regular Quercetin	Glibenclamide-induced INS-1 cells, H_2_O_2_-induced β-cells	↑ ERK 1/2 pathway	↑ Insulin secretion, Protection of INS-1 pancreatic β-cells	(36)
	Regular Quercetin	STZ-induced diabetic rats	↑ VEGF and VEGFR2	Repair of pancreatic islet β-cells	(37)
	Regular Quercetin	INS-1 cell line and isolated rat islets	↑ Ca^2+^ signaling pathway	↑ Insulin secretion	(38)
	Regular Quercetin	Male C57BLKS/J db/db mice, PA-treated MIN-6 cells	—	↑ Insulin secretion	(39)
	Regular Quercetin	—	↓ α-amylase, intestinal α-glucosidase	Inhibition of postprandial hyperglycemia	(40)
	Regular Quercetin	Sprague-Dawley rats	↑ CaMKKβ/AMPK pathway, insulin signaling; ↑ p-Akt, p-GSK-3β	Enhanced glucose utilization; ↑ Glucokinase (GCK)	(41)
	Regular Quercetin	Male Wistar rats	↑ LRP1, GLUT1, GLUT2, GLUT3, GLUT4	↑ Insulin sensitivity; ↓ Brain glucose	(42)
	Regular Quercetin	HFD/STZ-induced male albino rats	↓ ER stress, oxidative stress	↓ β-cell apoptosis, ↓ IR	(43)
	Regular Quercetin	High-fructose diet-induced rats	↑ Insulin receptor (InsR), Akt	↓ IR	(44)
	Regular Quercetin	TNF-α-induced human primary adipocytes	↓ IRS-1 serine phosphorylation, PTP1B	↓ IR	(45)
	Regular Quercetin	L6 myotube cells	↑ AMPK, p38	↓ IR	(46)
	Regular Quercetin	Sucrose- and maltose-induced rats	↓ α-glucosidase	↑ Insulin sensitivity, improved insulin signaling; ↓ Blood glucose	(47)
	Regular Quercetin	STZ-induced diabetic cataract (DC) rats	Binding to AR active sites (Tyr48, Gln49, Trp111)	↓ Aldose reductase (AR) activity	(48)
	Regular Quercetin	STZ-induced rats	↓ TGF-β2/PI3K/Akt	↓ AR activity, ↓ AGEs	(49)
	Regular Quercetin	High glucose-induced HUVECs	Inhibition of PARP-1 overactivation	↓ NAD^+^ depletion	(50)
	Regular Quercetin	Male C57BLKS/J db/db mice	↑ SIRT1, ↓ NLRP3	↓ ASC, cleaved Caspase-1, IL-1β, IL-18, blood glucose	(51)
	Regular Quercetin	HFD-induced C57BL/6J mice	—	Remodeling of WAT, ↓ adipose tissue expansion and inflammation	(52)
	Regular Quercetin	High-fructose diet-induced rats	↓ AMPK/TXNIP, ↓ NF-κB, NLRP3	Improved insulin signaling defects	(44)
	Regular Quercetin	High glucose-induced HepG2 cells	↑ NAD^+^/NADH, ↑ PGC-1α transcription	↓ ROS	(53)
	Quercetin liposomes	STZ-induced male Sprague-Dawley rats	—	↓ SOD, GSH-Px, MDA	(54)
	Regular Quercetin	STZ-induced Arbor Acre broiler chickens	Activation of PI3K/AKT pathway	↓ MDA, NO	(55)
	Quercetin-silver nanohydrogel	BB rats	—	Regulation of cytokines, growth factors, and proteases; promotion of skin wound healing	(56)
	Regular Quercetin	STZ-induced C57BL/6J mice	—	Inhibition of pancreatic iron deposition and β-cell ferroptosis	(57)
MAFLD	Regular Quercetin	Oleic acid-induced HepG2 cells	↑ Tyrosine phosphorylation	↑ Glucose uptake improved insulin resistance	(58)
	Regular Quercetin	FFA-induced HepG2 cells	↓ SREBP-1c, FAS gene expression	↓ IR, ↓ hepatic lipid accumulation	(59)
	Regular Quercetin	STZ-induced male SD rats	↓ LXR-α	↓ Hepatic lipogenesis; Inhibition of T2DM with MAFLD	(60)
	Regular Quercetin	HFD-fed male SD rats, FFA-induced HepG2 cells	↑ IRE1α-XBP1s	↑ VLDL synthesis, improved lipid metabolism	(61)
	Regular Quercetin	HFD-fed male SD rats	↑ NR1H4 (FXR)	Regulation of lipid metabolism, improved liver function, ↓ inflammation, ↓ and oxidative stress	(62)
	Regular Quercetin	Male C57BLKS/J db/db mice	↑ FXR1/TGR5	↓ IL-1β, IL-6, TNF-α; ↓ fat accumulation	(63)
	Regular Quercetin	MCD-induced C57BL/6J mice	↓ JNK, NF-κB	Inhibition of inflammation and fibrosis	(64)
	Regular Quercetin	HFD-induced gerbil model	↓ SIRT1, NF-κB p65, iNOS	↓ TNF-α, IL-6; ↓ fat accumulation	(65)
	Regular Quercetin	Intensive exercise-induced male BALB/C mice	Inhibition of PI3K and NF-κB pathways	↓ ER stress, ↓ inflammation	(66)
	Regular Quercetin	HFD-induced C57BL/6J mice	↑ Abundance of Verrucomicrobia, Akkermansia	Improved MAFLD pathology	(67)
	Regular Quercetin	HFD-induced C57BL/6J mice	↓ Desulfovibrio, Helicobacter; ↓ Firmicutes/Bacteroidetes ratio; ↑ Flavobacterium, Allobaculum, Clostridium, Proteobacteria	↑ SCFAs production; Restoration of intestinal barrier integrity; Improved MAFLD pathology	(68)
Obesity	Regular Quercetin	HFD-induced C57BL/6J mice	—	Inhibition of adipose tissue expansion, ↑ energy expenditure, and regulation of mitochondrial metabolism	(52)
	Regular Quercetin	3T3-L1 preadipocytes and mature adipocytes	↑ AMPK; ↓ ERK, JNK	↓ Adipogenic activity; Adipocyte apoptosis	(69)
	Regular Quercetin	3T3-L1 preadipocytes	—	Inhibition of adipocyte growth; Elimination of ROS	(70)
	Regular Quercetin	HFD-induced SD rats	Regulation of intestinal microbiota structure	↓ Lipid metabolism disorders	(71)
	Regular Quercetin	HFS-induced Wistar rats	↓ Firmicutes/Bacteroidetes ratio; ↓ Erysipelotrichaceae, Bacillus, *Eubacterium cylindroides*, *Wautersiella*; ↑ *Bacteroides vulgatus*, *Akkermansia muciniphila*	↓ Obesity	(72)
	Regular Quercetin	HFD-induced C57BL/6J mice	—	Improved abdominal obesity	(73)
	Regular Quercetin	HFD-induced C57BL/6J mice	↑ *Lachnospiraceae*, ILA/m6A/CYP8B1	Activation of FXR; ↓ Lipid accumulation	(74)
	Onion extract containing quercetin	HFD-induced SD rats	—	↓ Lipid accumulation in 3T3-L1 cells; ↓ intracellular TG	(75)
	Regular Quercetin	HFD-induced C57BL/6J mice	↓ Hepatic APOB expression	↓ Triglyceride content	(76)
	Regular Quercetin	Obese male Zucker rats and lean heterozygous littermates	—	↓ Body weight; ↓ TNF-α content	(77)
	Regular Quercetin	HFD-induced C57BL/6J mice	↓ CD68 and MCP-1 expression in IWAT and EWAT	↓ Obesity and inflammation	(52)
Atherosclerosis	Regular Quercetin	High-cholesterol diet-induced female SD rats; HUVECs	↓ TLR-NF-κB	↓ MCP-1, VCAM-1, ICAM-1; ↓ COX, 5-LOX, CRP activity	(78)
	Regular Quercetin	High fructose-induced C57BL/6 mice	↑ PI3K/Akt	↓ IL-1β, IL-6, IL-18, TNF-α; Inhibition of AS plaque formation	(79)
	Regular Quercetin	HFD/STZ-induced Wistar rats	↑ AMPK/SIRT1; ↓ NF-κB	↓ IL-1β, ↑ IL-10	(80)
	Regular Quercetin	HFD-induced ApoE^-^/^-^ mice	↓ Galectin-3, NLRP3	↓ IL-1β; ↓ inflammation	(81)
	Regular Quercetin	HFD-induced ApoE^-^/^-^ mice	Competitive binding to KEAP1 Arg483; ↑ Nrf2	Inhibition of macrophage pyroptosis	(82)
	Regular Quercetin	LPS or ox-LDL-stimulated RAW 264.7 cells	Inhibition of STAT3 pathway;	↓ MMP-1, SOCS3, IL-1α, IL-1β, IL-2, MCP-1, COX-2	(83)
	Regular Quercetin	HFD-induced ApoE^-^/^-^ mice	↑ NOX4; ↓ p47phox, p67phox	↓ Oxidative stress	(84)
	Regular Quercetin	HFD-induced ApoE^-^/^-^ mice	↓ NOX2 subunit p47phox expression	Inhibition of NOX-derived ROS; Improved AS plaques	(85)
	Regular Quercetin	oxLDL-treated HUVECs	↑ SIRT1/AMPK	↓ Oxidative damage	(86)
	Regular Quercetin	HFD-induced ApoE^-^/^-^ mice	↓ Serum isoprostane; ↑ eNOS activity; ↑ HO-1	Delayed AS lesions	(87)
	Regular Quercetin	Atherogenic diet-induced LDLR^-^/^-^ mice	—	↑ PON1 mRNA and serum levels; ↓ Aortic AS plaque area	(88)
	Regular Quercetin	HFD-induced ApoE^-^/^-^ mice	↑ ABCA1, LXRα; ↓ PCSK9	↓ AS plaque formation	(89)
	Regular Quercetin	oxLDL-treated RAW264.7 macrophages	Inhibition of LXRα/PCSK9-mediated ABCA1 expression	↓ Intracellular lipid accumulation; ↓ Foam cell formation	(90)
	Regular Quercetin	IFN-γ-induced THP-1 macrophages	↑ CD36, SR-A, LXRα gene expression	↑ Cholesterol uptake and efflux	(91)
	Regular Quercetin	HFD-induced ApoE^-^/^-^ mice; ox-LDL-induced HAECs	Regulation of p53 and mTOR pathways	Delayed HAEC senescence; ↓ Atherosclerotic lesions	(92)
	Regular Quercetin	HFD-induced ApoE^-^/^-^ mice	Inhibition of mTOR; ↑ LC3	↓ p53, p21 expression	(93)
	Regular Quercetin	ox-LDL-induced RAW264.7 macrophages	↑ LC3-II/I ratio, Beclin 1	Reversed pro-AS effects of 3-MA; ↓ p21, p16, SA-β-gal positive cells	(93)
	Regular Quercetin	Ldlr^-^/^-^ mice	↑ α-diversity; ↑ Actinobacteria, Bacteroidetes; ↓ Firmicutes; ↑ Akkermansia, Bacteroides, Parabacteroides, Ruminococcus	↓ Atherosclerotic lesions	(94)
Osteoporosis	Regular Quercetin	New Zealand white rabbit parietal bone	Binding to ERβ subtype; Activation of ER-mediated signaling	↑ Osteoblast proliferation and mineralization	(95)
	Regular Quercetin	C57BL/6 mouse BMSCs	↑ Runx2, OSX, OPN; Activation of BMP signaling	↑ BMSCs proliferation and osteogenic differentiation	(96)
	Regular Quercetin	BMSCs	Regulation of specific circRNAs, affecting miR-326-5p and mRNAs related to osteogenic/adipogenic differentiation	↑ BMSCs osteogenic capacity; ↓ Adipogenic differentiation	(97)
	Regular Quercetin	BMSCs	↑ H19/miR-625-5p axis; Activation of Wnt/β-catenin	↑ BMSCs osteogenic differentiation	(98)
	Regular Quercetin	Female SD rats (OVX model)	Restoration of RANKL/OPG balance	Inhibition of osteoclast activity; ↑ Bone healing	(99)
	Regular Quercetin	Female SD rats (OVX model)	Regulation of autophagy genes (LC3, BECN1) and apoptosis genes (Caspase-3)	Regulated osteocyte autophagy and apoptosis	(100)
	Regular Quercetin	Orchiectomy-induced C57BL/6 mice	↑ GPRC6A/AMPK; ↓ mTOR	↓ OP symptoms	(101)
	Regular Quercetin	Female SD rats (OVX model)	Regulation of gut microbiota-SCFA-inflammation axis	Regulation of microbiota structure; Improved inflammatory status	(102)
	Regular Quercetin	BMSCs	↓ NF-κB	↓ BMSCs inflammatory response; ↑ Osteogenic differentiation	(103)
	Regular Quercetin	Female SD rats (OVX model)	↓ NLRP3	↓ Inflammatory response	(104)
PCOS	Regular Quercetin	DHEA-induced SD rats	↓ TLR2-NF-κB	↓ IL-1β, IL-6, TNF-α	(105)
	Regular Quercetin	DHEA-induced SD rats	↑ GLUT4	↑ Hexokinase and glucokinase activity; ↓ IR and insulin levels	(106)
	Regular Quercetin	Letrozole-induced Wistar rats	↑ AMPK/SIRT-1	Improved lipid profile, sex hormone levels, and IR; Reversed abnormal adiponectin, visfatin, and vaspin levels	(107)
	Regular Quercetin	TP-induced SD rats	Binding to PI3K, blocking PI3K pathway	↓ Cyp17a1 gene expression; ↓ Cyp17a1 content	(108)
	Regular Quercetin	DHEA-induced Wistar rats	↑ Aromatase	Blocking androgen conversion to estrogen	(109)
	Regular Quercetin	Letrozole-induced SD rats	—	↓ Testosterone and estradiol levels; ↓ Ovarian and cystic follicle diameter	(110)
	Regular Quercetin	Letrozole-induced Parks mice	—	↑ Steroidogenesis: Regulation of folliculogenesis-related genes	(111)
Hyperuricemia	Regular Quercetin	—	Binding to XOD, inhibiting XOD activity	↓ Serum uric acid	(112)
	Regular Quercetin	Potassium oxonate-induced BALB/c mice	Binding to XOD, inhibiting XOD activity	↓ Serum uric acid	(113)
	Regular Quercetin	HUVECs	Inhibition of ADA, PNP, and XOR activity	↓ Serum uric acid	(114)
	Regular Quercetin	Fructose-induced SD rats	↑ OAT, UAT; ↓ GLUT9, RST	↓ Serum BUN, Scr; Promotion of uric acid excretion	(115)
	Regular Quercetin	Fructose-induced SD rats	Improvement of UAT, GLUT9, OAT1, mOCTs expression; ↓ UMOD	↑ Uric acid excretion	(115)
	Regular Quercetin	CdCl_2_-induced male SD rats	↑ NAG/Cr, RBP, β2-MG	↓ XOR activity	(116)
Hyperlipidemia	Regular Quercetin	HCD-induced SD rats	—	↓ Serum TC, hepatic TC, hepatic TG, LDL-C; ↑ HDL-C	(117)
	Regular Quercetin	HCD-induced SD rats	—	↓ Serum TG, TC, VLDL	(117)
	Regular Quercetin	High-cholesterol-induced Wistar rats	↓ NPC1L1 expression	Inhibition of intestinal cholesterol absorption	(118)
	Regular Quercetin	THP-1 cells	↑ PPARγ, LXRα; ↑ ABCA1 gene expression	↑ Cholesterol efflux	(119)
Gouty Arthritis	Regular Quercetin	Acetic acid-induced Swiss albino mice	Inhibition of COX-2 protein expression	Inhibition of xanthine and hypoxanthine conversion to uric acid	(120)
	Regular Quercetin	MSU-induced SD rats	↓ IL-6, IL-17A, IL-17F (IL-17 pathway)	↓ Ankle bone destruction	(121)

↑ = Upregulation/Promotion/Increase;↓ = Downregulation/Inhibition/Decrease;— = Not reported/Not applicable.

### 6.1 Diabetes mellitus

Diabetes mellitus (DM) is a metabolic disorder characterized by impaired insulin secretion and/or IR, leading to chronic hyperglycemia and disturbances in carbohydrate, lipid, and protein metabolism (122). Prolonged hyperglycemia can induce various systemic complications, including vascular disorders, renal dysfunction, retinopathy, and peripheral neuropathy. Once developed, these complications are largely irreversible and represent major causes of disability and mortality in DM patients. Globally, DM affected approximately 537 million individuals in 2021, with projections estimating a 46% increase to 783 million cases by 2045, the majority being T2DM (40). Accumulating evidence indicates that quercetin exhibits significant anti-diabetic properties through multiple mechanisms, including promotion of insulin secretion, improvement of IR, maintenance of glucose homeostasis, as well as anti-inflammatory and antioxidant effects.

Quercetin enhances insulin secretion via ERK1/2 activation, VEGF-mediated β-cell protection, and Ca^2+^ signaling, with glucose-dependent effects (41). The compound also modulates calcium signaling pathways to facilitate insulin secretion (42). Notably, quercetin exhibits glucose-dependent insulinotropic effects, showing minimal stimulation of insulin secretion in the absence of glucose but inducing a threefold increase at glucose concentrations of 8.3 mmol/L (43). Dose-response studies further reveal that quercetin effectively reduces serum glucose levels at doses ranging from 10 to 50 mg/kg in animal models (44).

In glucose metabolism regulation, quercetin inhibits key carbohydrate-digesting enzymes, including pancreatic α-amylase and intestinal α-glucosidase, thereby slowing starch hydrolysis and reducing intestinal glucose absorption to attenuate postprandial hyperglycemia (45). Simultaneously, it enhances peripheral glucose uptake and utilization through activation of the CaMKKβ/AMPK pathway and insulin signaling cascades in muscle tissues (123). In hepatic glucose metabolism, quercetin upregulates glucokinase (GCK) expression by increasing phosphorylation of glycogen synthase kinase-3β (GSK-3β) and protein kinase B (Akt), thereby promoting hepatic glycogen synthesis. Furthermore, quercetin improves cerebral glucose metabolism by enhancing the expression of insulin signaling components, low-density lipoprotein receptor-related protein 1 (LRP1), and glucose transporters (GLUT1-4), effectively ameliorating hyperglycemic conditions in neural tissues (46).

The anti-diabetic effects of quercetin are further supported by its ability to mitigate chronic low-grade inflammation and oxidative stress, both of which play crucial roles in DM pathogenesis (124). By suppressing pro-inflammatory cytokines such as TNF-α and IL-6 while inhibiting NF-κB signaling pathways, quercetin alleviates IR. Its potent antioxidant properties protect β-cells from oxidative damage and improve mitochondrial function (47). These comprehensive mechanisms demonstrate quercetin’s potential as a multi-target therapeutic agent for DM management, though further clinical studies are warranted to validate its efficacy and safety in human patients (125).

Quercetin has been demonstrated to improve IR through multiple mechanisms (126). Animal studies show that quercetin alleviates pancreatic endoplasmic reticulum stress and oxidative stress, thereby reducing β-cell apoptosis and significantly improving IR in diabetic rats. It modulates hypothalamic insulin signaling by upregulating phosphorylation of insulin receptors (InsRs) and Akt. *In vitro* evidence indicates quercetin suppresses TNF-α-induced IR in human primary adipocytes by inhibiting IRS-1 serine phosphorylation and PTP1B gene expression. Furthermore, quercetin enhances glucose uptake in skeletal muscle cells via activation of the AMPK pathway and its downstream target p38 (127).

The compound also exhibits potent α-glucosidase inhibitory activity (128). By competitively binding to key active sites through hydrogen bond formation, quercetin effectively inhibits α-glucosidase activity, thereby reducing postprandial hyperglycemia. This mechanism parallels that of clinical α-glucosidase inhibitors, suggesting quercetin’s potential as a dietary supplement for diabetes management. Experimental data demonstrate its ability to improve insulin sensitivity and signaling in mice while ameliorating fasting hyperglycemia (48, 129).

Quercetin functions as an aldose reductase inhibitor (ARI) by binding to the enzyme’s active sites and suppressing the polyol pathway. This action prevents sorbitol accumulation and subsequent diabetic complications, including cataracts, retinopathy, and neuropathy (49, 50, 130). The compound also inhibits TGF-β2/PI3K/Akt signaling to reduce advanced glycation end product (AGE) formation (51). Notably, quercetin preserves cellular NAD+ levels by preventing PARP-1 overactivation, offering protection against diabetic complications (52, 53, 131).

Anti-inflammatory effects of quercetin include suppression of NLRP3 inflammasome activation and NF-κB signaling pathway (132). These actions lead to reduced expression of pro-inflammatory cytokines (IL-6, TNF-α, PAI-1) and amelioration of diabetic complications such as encephalopathy and nephropathy (54). The compound also promotes white adipose tissue remodeling and improves hypothalamic insulin signaling through AMPK/TXNIP modulation (47, 133).

Quercetin’s antioxidant properties are manifested through multiple pathways. It scavenges reactive oxygen species (ROS) directly, chelates transition metal ions, and enhances cellular antioxidant defenses by upregulating SOD and GSH-Px activities while decreasing Malondialdehyde (MDA) levels (55, 56, 134, 135). The compound protects mitochondrial function by maintaining NAD+/NADH balance and activating PGC-1α, thereby preserving β-cell function (57, 136–138).

Additional therapeutic benefits include wound healing promotion through the regulation of growth factors and cytokines, as well as inhibition of pancreatic iron deposition and ferroptosis (139, 140). The multifaceted mechanisms of quercetin involve modulation of various signaling pathways (ERK1/2, VEGF, PI3K/AKT, NF-κB), metabolic enzymes (α-amylase, α-glucosidase, AR), and molecular targets (GLUTs, InsRs, NLRP3), collectively contributing to its antidiabetic effects. These findings highlight quercetin’s potential as a comprehensive therapeutic agent for diabetes and its complications.

### 6.2 Metabolic dysfunction-associated fatty liver disease

MAFLD (formerly NAFLD), defined by hepatic steatosis with metabolic dysfunction, affects ~25% of adults globally (141). In 2023, an international expert consensus proposed renaming NAFLD to MAFLD, defining it as hepatic steatosis (confirmed by imaging or biopsy) accompanied by at least one of the following: type 2 diabetes, obesity, or metabolic dysregulation (142). Currently representing the most prevalent chronic liver disease worldwide, with an adult prevalence of approximately 25%, MAFLD demonstrates strong associations with hepatic decompensation, hepatocellular carcinoma, and cardiorenal metabolic diseases (143). Despite four decades of research, effective pharmacological treatments remain limited, with experimental therapies like resmetirom and semaglutide showing promise but not yet widely implemented (58, 106). Retrospective analyses indicate quercetin may ameliorate MAFLD through multiple pathways, including lipid metabolism regulation, anti-inflammatory effects, and antioxidant activity (59, 144).

Quercetin improves IR in MAFLD by enhancing hepatic glucose utilization and reducing lipid accumulation (60). It upregulates GLUT4 expression while increasing hexokinase and glucokinase activity, significantly lowering IR and insulin levels in animal models (145). *In vitro* studies demonstrate that quercetin promotes insulin signaling through enhanced tyrosine phosphorylation, facilitating glucose uptake (146). The compound also suppresses sterol regulatory element-binding protein-1c (SREBP-1c) and fatty acid synthase (FAS) expression, thereby ameliorating hepatic lipid accumulation and IR (61, 62).

The regulation of lipid metabolism by quercetin involves multiple mechanisms. It modulates cholesterol homeostasis through mTOR/YY1 signaling pathway inhibition and direct activation of cholesterol 7α-hydroxylase (CYP7A1) transcription, promoting cholesterol conversion to bile acids (147, 148). Quercetin reduces hepatic lipid synthesis by downregulating liver X receptor-α (LXR-α) expression and decreases oxidized low-density lipoprotein (OX-LDL) accumulation (63). Activation of the IRE1α-XBP1s signaling pathway enhances very low-density lipoprotein (VLDL) assembly and lipophagy, while upregulation of bile acid receptor NR1H4 alleviates hepatic steatosis (64, 65). Emerging evidence suggests quercetin’s involvement in linoleic acid and glycerophospholipid metabolism, though detailed mechanisms require further investigation (65, 66).

Anti-inflammatory effects of quercetin in MAFLD include TLR/NF-κB pathway inhibition, resulting in reduced pro-inflammatory cytokine production and improved pancreatic β-cell function (149, 150). The compound activates FXR/TGR5 signaling while suppressing JNK and NF-κB pathways, thereby attenuating hepatic inflammation and fibrosis (67, 151). Quercetin decreases SIRT1, NF-κB p65, and inducible nitric oxide synthase (iNOS) expression, subsequently lowering serum TNF-α and IL-6 levels (151). *In vitro* studies confirm its ability to reduce IL-8 expression and mitigate lipid-induced inflammatory damage. Additionally, quercetin alleviates endoplasmic reticulum stress through PI3K/NF-κB pathway modulation (68, 146).

Quercetin’s antioxidant properties in MAFLD involve CYP2E1 suppression and nuclear factor erythroid 2-related factor 2 (Nrf2) activation (152). The compound demonstrates superior antioxidant capacity compared to pioglitazone and citric acid in experimental models (153). By upregulating Nrf2 expression and its downstream target glutathione peroxidase 4 (GPX4), quercetin enhances cellular antioxidant defenses in MAFLD progression (154).

The modulation of gut microbiota represents another therapeutic mechanism of quercetin in MAFLD. It increases Verrucomicrobiota, Verrucomicrobiae, and Akkermansia abundance while reducing Desulfovibrio and Helicobacter populations (155). Quercetin restores the Firmicutes/Bacteroidetes ratio and enhances Flavobacterium, Clostridium, and Proteobacteria abundance, concurrently improving short-chain fatty acid production and intestinal barrier integrity (156). Fecal microbiota transplantation studies confirm that these microbial changes confer MAFLD resistance in recipient animals (157).In summary, quercetin exerts anti-MAFLD effects through regulation of multiple targets.

### 6.3 Obesity

Obesity, defined by the World Health Organization as excessive fat accumulation that poses health risks, results from adipocyte hyperplasia and hypertrophy through the differentiation of preadipocytes into mature fat cells (158). Recognized as a major risk factor for chronic diseases, including metabolic syndrome, type 2 diabetes, cardiovascular diseases, stroke, and certain cancers, obesity represents a significant public health challenge (159). Current therapeutic approaches encompass dietary modification, exercise, behavioral interventions, pharmacotherapy, and bariatric surgery, yet long-term efficacy remains limited (69, 160, 161). This underscores the urgent need for effective anti-obesity strategies. Emerging evidence indicates that quercetin may ameliorate obesity through multiple mechanisms involving adipocyte function modulation and inflammatory regulation (70, 162).

Quercetin exerts inhibitory effects on adipose tissue growth through multifaceted mechanisms (133). It suppresses high-fat diet-induced white adipose tissue expansion while enhancing energy expenditure and mitochondrial biogenesis (71). During adipogenesis, quercetin significantly downregulates mRNA expression of key lipogenic genes. The activation of the AMP-activated protein kinase (AMPK) pathway represents a crucial mechanism whereby quercetin increases phosphorylated AMPK levels and inactivates acetyl-CoA carboxylase (ACC), consequently reducing fatty acid synthesis while promoting fatty acid oxidation (72). Additionally, quercetin enhances serotonin (5-HT) synthesis in chemosensory neurons (ADF) of obese mice, thereby stimulating lipolysis and fatty acid β-oxidation (73). In 3T3-L1 preadipocytes, quercetin demonstrates remarkable anti-adipogenic effects with 71.5% growth inhibition, attributable to its potent antioxidant capacity and reactive oxygen species (ROS) scavenging properties (163).

The modulation of gut microbiota by quercetin contributes to its anti-obesity effects. Experimental studies reveal that quercetin ameliorates lipid metabolic disorders in obese rats while favorably altering gut microbial composition (74). Specifically, it reduces the Firmicutes/Bacteroidetes ratio and suppresses obesity-associated bacteria, including Erysipelotrichaceae, Bacillus, Eubacterium cylindroides, and Bilophila wadsworthia, while increasing beneficial species such as Bacteroides vulgatus and Akkermansia muciniphila (75). Notably, quercetin enhances short-chain fatty acid production concurrent with microbial modulation (76). Further investigations demonstrate its ability to ameliorate monosodium glutamate-induced gut dysbiosis, mitigate hypothalamic damage, and downregulate hepatic retinol saturase (RetSat) expression, collectively improving abdominal obesity. Recent findings highlight quercetin’s capacity to enrich Akkermansia muciniphila populations, which promote indole-3-lactic acid (ILA) synthesis (164). ILA subsequently activates the FTO/m6A/YTHDF2 pathway to upregulate CYP8B1 expression, facilitating cholesterol conversion to cholic acid (CA). This bile acid then inhibits lipid accumulation through adipose tissue farnesoid X receptor (FXR) activation, establishing a novel anti-obesity mechanism (165).

Quercetin reduces adipocyte accumulation and promotes apoptosis through multiple pathways. It significantly decreases intracellular triglyceride (TG) content in 3T3-L1 preadipocytes by downregulating hepatic apolipoprotein B (APOB) expression and enhancing subcutaneous white adipose tissue uptake of TG-derived fatty acids, promoting adipose tissue browning (166). Dietary quercetin supplementation reduces body weight and visceral fat deposition in obese rats, accompanied by downregulation of PPARγ and C/EBPα mRNA expression (77). The compound suppresses lipogenic genes, including FAS and ACC, while upregulating CPT-1α and UCP-1, indicating enhanced lipid catabolism and suppressed lipogenesis (166). Mechanistic studies reveal that quercetin induces adipocyte apoptosis through mitochondrial membrane potential reduction, PARP and Bcl-2 downregulation, and activation of caspase-3 and Bak. Furthermore, it inhibits phosphodiesterase (PDE) to accelerate lipid degradation in adipocytes (167). In both 3T3-L1 cells and mature adipocytes, quercetin activates AMPK signaling while modulating ERK and JNK pathways to exert anti-adipogenic and pro-apoptotic effects (71).

The anti-inflammatory properties of quercetin ameliorate obesity-associated low-grade inflammation. In the obese state, hypertrophic adipocytes secrete proinflammatory cytokines including IL-6, MCP-1, and TNF-α (168, 169). Quercetin treatment reduces body weight in Zucker diabetic fatty rats while significantly suppressing TNF-α secretion (170). It downregulates the expression of chronic inflammation markers (Cd68, MCP-1) in both inguinal and epididymal white adipose tissues, although precise mechanisms require further elucidation (133).

In summary, quercetin combats obesity through comprehensive regulation of molecular targets. Its modulation of gut microbiota composition (Firmicutes/Bacteroidetes ratio, increased Akkermansia and Bacteroides, decreased Firmicutes/Bacteroidetes ratio) and microbial metabolites (SCFAs, CA) further contributes to its therapeutic efficacy against obesity.

### 6.4 Atherosclerosis

Atherosclerosis (AS) is a chronic inflammatory disease characterized by lipid accumulation and inflammatory cell infiltration in medium and large arterial walls, representing a leading cause of global morbidity and mortality (171). The pathogenesis involves both traditional risk factors (hyperlipidemia, hypertension) and emerging contributors (sleep disorders, gut dysbiosis, air pollution) (78). In 2022 alone, cardiovascular diseases (CVD) secondary to AS caused approximately 19.8 million deaths worldwide, with over 34% occurring prematurely before age 70 (79). Current pharmacotherapies primarily target lipid modulation and inflammation (IL-6/IL-1β inhibition) (80). Growing evidence demonstrates that quercetin, a dietary flavonoid, exerts multi-target anti-atherosclerotic effects through anti-inflammatory, antioxidant, and metabolic regulatory mechanisms (172).

Quercetin modulates AS-related inflammation through multiple pathways. It suppresses NF-κB activation by inhibiting TLR2/TLR4 signaling, thereby reducing oxLDL-induced expression of MCP-1, VCAM-1, and ICAM-1 in HUVECs, while decreasing COX, 5-LOX, and CRP activity. The compound antagonizes NF-κB-mediated cytokine production (IL-1β, IL-6, IL-18, TNF-α) via PI3K/Akt pathway activation, ameliorating LPS-induced Zal FMC inflammation and high-fructose diet-induced plaque formation (82). Through AMPK/SIRT1/NF-κB signaling, quercetin lowers IL-1β while elevating IL-10 in AS models. It also inhibits dendritic cell maturation by suppressing Src/PI3K/Akt/NF-κB cascades, evidenced by reduced IL-6/IL-12 and increased IL-10 levels (83). Notably, quercetin blocks NLRP3 inflammasome activation by interfering with galectin-3 binding in plaque macrophages, consequently reducing IL-1β secretion. Competitive binding at KEAP1-Arg483 promotes Nrf2 activation, inhibiting NLRP3-mediated pyroptosis in ApoE-/- mice (79, 84, 85). As a STAT3 inhibitor, quercetin downregulates MMP-1 and SOCS3 transcription, attenuating macrophage expression of IL-1α, IL-1β, IL-2, MCP-1, and COX-2 (81, 86).

The compound demonstrates potent redox-modulating properties. It counteracts NOX4 downregulation in ApoE-/- mice aortas while normalizing p47phox/p67phox overexpression to alleviate oxidative stress (172). Through SIRT1/AMPK pathway activation, quercetin suppresses oxLDL-induced NOX4 expression in HUVECs (87, 88). Antioxidant enzyme enhancement includes HO-1 upregulation, which reduces isoprostane F2α and boosts eNOS activity. Quercetin increases hepatic PON1 mRNA and serum protein levels while preventing PON2 decline in ApoE-/- mice, collectively improving antioxidant defenses and reducing plaque burden (89–91, 173).

Lipid metabolic regulation involves cholesterol homeostasis modulation. Quercetin upregulates ABCA1/LXRα while downregulating PCSK9, inhibiting foam cell formation in RAW264.7 macrophages (92). It balances cholesterol flux by modulating CD36, SR-A, and LXRα expression in THP-1 macrophages (93). Anti-senescence effects manifest through p53/mTOR pathway inhibition, evidenced by reduced SA-β-gal positivity, p21/p16 expression, and mitochondrial dysfunction in oxLDL-treated HAECs (94, 174). Quercetin rescues autophagy impairment (increased LC3-II/I ratio and Beclin 1) via MST1 regulation, counteracting 3-MA-induced pro-atherogenic effects (175, 176).

Additional mechanisms include metal ion chelation (inhibiting Fenton reaction-derived radicals) and gut microbiota modulation (increased α-diversity with elevated Actinobacteria/Bacteroidetes and Akkermansia/Bacteroides/Parabacteroides/Ruminococcus, decreased Firmicutes/Lactobacillus) (14, 177).

In summary, quercetin targets TLR2/4-NF-κB, AMPK/SIRT1, PI3K/Akt, mTOR, NLRP3, KEAP1/Nrf2, STAT3, VCAM-1/ICAM-1, HO-1/eNOS, PON1/2, and inflammatory cytokines (IL-1β/6/18, TNF-α), while modulating gut microbial composition (Firmicutes/Bacteroidetes ratio, Akkermansia, Bacteroides, Parabacteroides, Ruminococcus, Lactobacillus) to combat AS progression.

### 6.5 Osteoporosis

Osteoporosis is a systemic skeletal disease characterized by decreased bone density, deterioration of bone tissue microstructure, and increased bone fragility (178). The imbalance between bone formation and resorption, along with impaired osteogenic differentiation of bone marrow mesenchymal stem cells (BMSCs), plays a crucial role in its pathogenesis (179). Currently affecting approximately 18.3% of the global population, the prevalence of osteoporosis continues to rise with aging demographics. While existing treatments, including hormone therapy, antiresorptive agents, and bone-forming medications, can alleviate symptoms to some extent, they are often associated with adverse effects and variable individual responses (95, 96). Research indicates that quercetin may improve osteoporosis through multiple mechanisms, including anti-inflammatory, antioxidant, and bone cell metabolism regulation (97).

Quercetin exhibits estrogen-like effects that are particularly relevant for postmenopausal osteoporosis. It binds specifically to both estrogen receptor α (ERα) and estrogen receptor β (ERβ), mimicking the physiological effects of estrogen on bone cells (9). Through interaction with ERβ, quercetin activates estrogen receptor-mediated signaling pathways that promote osteoblast proliferation and mineralization (180). This includes upregulation of key osteogenic markers such as Runx2, OSX, and OPN, as well as activation of bone morphogenetic protein (BMP) signaling to enhance BMSC proliferation and osteogenic differentiation (181). Furthermore, quercetin induces conformational changes in BMP-2 by altering the microenvironment around tyrosine residues, thereby increasing its thermal stability and osteogenic activity (182). Additional studies demonstrate quercetin’s ability to regulate specific circular RNAs that influence the interaction between miR-326-5p and mRNAs related to osteogenic and adipogenic differentiation, effectively reversing the decreased osteogenic capacity and enhanced adipogenesis of BMSCs caused by ERα deficiency (183, 184).

The antioxidant properties of quercetin play a significant role in combating osteoporosis, as oxidative stress represents a key pathogenic factor that damages bone cells and disrupts bone microstructure (98). Quercetin effectively mitigates oxidative damage to the skeletal system through free radical neutralization and inhibition of reactive oxygen species production (185, 186). Its influence on BMSC differentiation is particularly noteworthy, as these cells possess multipotent differentiation potential critical for maintaining bone health (99). By activating the Wnt/β-catenin signaling pathway through upregulation of the H19/miR-625-5p axis, quercetin promotes osteogenic differentiation of BMSCs. For osteoclasts, which originate from hematopoietic stem cells and possess potent bone resorptive capacity, quercetin exerts dose-dependent inhibitory effects on their activity through modulation of the RANKL/OPG signaling pathway (101, 102). The compound also suppresses osteoclast differentiation via the c-fos/cSrc/NFATC1 signaling pathway while simultaneously promoting osteoblast proliferation through BMP-2/Smad4 signaling activation (187). Additional mechanisms include regulation of autophagy-related genes (LC3, BECN1) and apoptosis-related genes (Caspase-3), as well as modulation of glucose and lipid metabolism through the GPRC6A/AMPK/mTOR pathway in testosterone-deficient conditions (100). Emerging evidence also suggests quercetin may improve bone loss in ovariectomized rats by regulating the gut microbiota-short-chain fatty acid-inflammatory signaling axis, although the precise mechanisms require further investigation (103, 104, 188, 189).

The anti-inflammatory effects of quercetin contribute significantly to its anti-osteoporotic properties (190). Excessive production of inflammatory cytokines such as TNF-α, IL-1β, and IL-6 accelerates bone destruction and disrupts bone remodeling balance (191). Quercetin demonstrates remarkable potential in mitigating osteoporosis-related inflammation by suppressing both the production and release of inflammatory mediators (192). It alleviates inflammatory responses in BMSCs through inhibition of the NF-κB signaling pathway, counteracting cellular senescence while promoting osteogenic differentiation. Additional anti-inflammatory mechanisms involve the regulation of the NLRP3 inflammasome to reduce the expression of osteoporosis-related inflammatory mediators (193). Quercetin also reverses TNF-α-induced downregulation of the long non-coding RNA Malat1, thereby enhancing osteogenic activity (105).

In summary, quercetin exerts anti-osteoporotic effects through comprehensive regulation of multiple targets, including ERα, ERβ, BMP signaling, BMP-2, Wnt/β-catenin signaling, RANKL/OPG pathway, c-fos/cSrc/NFATC1 signaling, GPRC6A/AMPK/mTOR pathway, H19/miR-625-5p axis, NF-κB, NLRP3, and various inflammatory mediators (TNF-α, IL-1β, IL-6). These multifaceted actions position quercetin as a promising therapeutic candidate for osteoporosis treatment.

### 6.6 Polycystic ovary syndrome

Polycystic ovary syndrome (PCOS) is a prevalent reproductive endocrine disorder affecting approximately 11-13% of women of reproductive age worldwide (194). Characterized by ovarian enlargement, hyperandrogenism, hirsutism, acne, menstrual irregularities, and ovulatory dysfunction, PCOS often leads to long-term complications, including IR and compensatory hyperinsulinemia, posing significant health and economic burdens globally (195). Current pharmacological interventions typically target single pathological aspects of PCOS and are frequently associated with adverse effects, highlighting the need for safer multi-target therapeutic agents (107). Emerging evidence demonstrates that quercetin, a natural flavonoid, exerts comprehensive therapeutic effects by modulating multiple key pathways in PCOS pathogenesis (196).

Quercetin effectively ameliorates chronic low-grade inflammation, a central feature of PCOS pathophysiology marked by elevated leukocyte counts and increased inflammatory markers, including CRP, IL-6, IL-18, MCP-1, and MIP-1α (197). Experimental studies confirm quercetin’s ability to significantly reduce serum levels of pro-inflammatory cytokines such as IL-6 and TNF-α in PCOS rat models, thereby attenuating systemic inflammatory responses (149). The compound’s anti-inflammatory mechanism involves suppression of the TLR/NF-κB signaling pathway in ovarian tissue, which not only improves the inflammatory microenvironment but also protects pancreatic β-cell function while normalizing serum insulin and fasting glucose levels.

The therapeutic potential of quercetin extends to improving IR, a hallmark metabolic disturbance in PCOS (145). Through upregulation of GLUT4 expression and enhancement of hepatic hexokinase and glucokinase activity, quercetin significantly ameliorates IR and hyperinsulinemia in PCOS models (198). Its metabolic regulatory effects are further mediated via activation of the AMPK/SIRT-1 pathway, which corrects dyslipidemia and normalizes serum sex hormone levels while restoring physiological concentrations of adipokines, including adiponectin, visfatin, and resistin (199). Notably, quercetin suppresses elevated levels of pro-adipogenic factors angptl-4 and adipsin in adipocytes, an effect closely associated with AMPK activation (109, 200).

Quercetin demonstrates remarkable efficacy in addressing hyperandrogenism (FOH) by modulating hypothalamic-pituitary-gonadal axis dysfunction (108). The compound’s glycoside core competitively binds to PI3K, completely inhibiting PI3K/Akt signaling and downregulating Cyp17a1 expression, thereby blocking progesterone conversion to androgens (201). Animal studies reveal that quercetin increases ovarian aromatase content in DHEA-induced PCOS rats, preventing androgen-to-estrogen conversion (110). These mechanisms collectively counteract excessive ovarian androgen production and secretion (111, 202).

For ovulatory dysfunction, quercetin restores normal folliculogenesis by modulating steroidogenesis in letrozole-induced PCOS rats (203). Treatment significantly reduces cystic follicle numbers while increasing primordial and primary follicle counts, accompanied by normalized theca and granulosa cell layers (204). These morphological improvements correlate with hormonal changes, including decreased testosterone and estradiol levels, demonstrating comparable efficacy to metformin. Quercetin enhances corpus luteum formation and oocyte regeneration capacity, potentially through regulation of steroidogenic and folliculogenic gene expression patterns that create favorable conditions for ovulation (205).

In summary, quercetin exerts multi-target therapeutic effects in PCOS through comprehensive modulation of TLR/NF-κB and PI3K/Akt signaling pathways, GLUT4 expression, GnRH sensitivity, AMPK/SIRT-1 activation, and regulation of various adipokines (angptl-4, adipsin) and reproductive hormones (LH, FSH, DHEA, SHBG, progesterone, testosterone, estradiol). Its simultaneous anti-inflammatory action through reduction of CRP, IL-6, IL-18, MCP-1, MIP-1α, and TNF-α further contributes to the amelioration of PCOS pathology. These multifaceted pharmacological properties position quercetin as a promising candidate for PCOS treatment.

### 6.7 Hyperuricemia

Uric acid, the end product of purine metabolism, is primarily produced in the liver, muscles, and adipose tissue, with approximately 75% excreted through renal pathways in healthy individuals (206). The delicate equilibrium between uric acid production, renal excretion, and intestinal absorption is crucial for maintaining physiological homeostasis. Disruption of this balance can lead to HUA, a condition characterized by excessive uric acid accumulation that not only causes painful crystal deposition in joints and soft tissues but is also closely associated with gouty arthritis, renal disorders, cardiovascular diseases, and hypertension (207). With a prevalence of 20.1% in the United States and 14% in mainland China, HUA has emerged as the second most common metabolic disorder after type 2 diabetes (208). As the primary precursor to gout, HUA contributes significantly to global disease burden, with current estimates indicating 5.8 million gout cases worldwide in 2020, projected to reach 95.8 million by 2050 (112, 114). Research demonstrates that quercetin exerts therapeutic effects against HUA and gouty arthritis through dual mechanisms of reducing uric acid production and enhancing urate excretion (113).

Quercetin effectively inhibits uric acid biosynthesis by targeting key enzymes in the purine catabolic pathway. Xanthine oxidase (XOD), the rate-limiting enzyme that catalyzes the conversion of xanthine to uric acid, is competitively inhibited through quercetin’s direct binding, which alters the enzyme’s secondary structure and disrupts its active site formation (209). Experimental evidence shows quercetin significantly suppresses serum uric acid levels in HUA mice by downregulating XOD expression (210). *In vitro* studies using human umbilical vein endothelial cells reveal quercetin’s capacity to counteract uric acid-induced elevation of adenosine deaminase (ADA), purine nucleoside phosphorylase (PNP), and xanthine oxidoreductase (XOR) activities, thereby reducing cellular uric acid concentrations (115, 116, 211).

The renal excretion of urates is enhanced by quercetin through modulation of specialized transport systems. organic anion/cation transporters (OATs, OCTs) and urate transporters (212). These changes are accompanied by reduced serum levels of blood urea nitrogen (BUN) and creatinine (Scr), indicating preserved renal function (213).

Additional nephroprotective effects involve the AMPK-PPARα/PGC-1β signaling pathway activation, which ameliorates renal lipid accumulation and decreases XOR activity. Quercetin treatment normalizes urinary biomarkers, including N-acetyl-β-D-glucosaminidase/creatinine ratio (NAG/Cr), retinol-binding protein (RBP), and β2-microglobulin (β2-MG), reflecting improved tubular integrity and reduced renal toxicity (214). Through these multifaceted actions on enzymatic inhibition and transport regulation, quercetin demonstrates comprehensive therapeutic potential against HUA and its complications.

### 6.8 Hyperlipidemia

Hyperlipidemia, characterized by elevated serum TG and TC, has become a significant global health concern, particularly prevalent among obese and diabetic populations (215). This metabolic disorder demonstrates a worrying trend of increasing incidence among younger individuals and serves as a major risk factor for various chronic conditions, including hypertension, fatty liver disease, cirrhosis, peripheral vascular diseases, ischemic cerebrovascular diseases, and pancreatitis (216, 217). Cardiovascular diseases claim approximately 17.9 million lives annually worldwide, with hyperlipidemia patients facing twice the risk compared to normolipidemic individuals (117). Current pharmacological interventions, such as fibrates, statins, bile acid sequestrants, niacin, and cholesterol absorption inhibitors, while widely used, are associated with adverse effects, including hepatotoxicity, neurological complications, and increased diabetes risk during long-term therapy, necessitating the development of safer alternative treatments (118). Research indicates that quercetin effectively ameliorates dyslipidemia through multiple mechanisms (218).

Experimental studies demonstrate quercetin’s capacity to significantly reduce serum TC (31%), hepatic TC, hepatic TG, and low-density lipoprotein cholesterol (LDL-C) levels while elevating high-density lipoprotein cholesterol (HDL-c) concentrations in hyperlipidemic rats (219). The compound effectively normalizes serum VLDL levels and improves overall lipid profiles. At the molecular level, quercetin downregulates the expression of intestinal cholesterol transporter Niemann-Pick C1-like 1 (NPC1L1), thereby specifically inhibiting dietary cholesterol absorption (119, 212). Comprehensive modulation of lipid metabolism is achieved through quercetin’s influence on multiple gene expressions, including Fnta, Pon1, Aldh1b1, Abcg5, Apoa4, Acaca, FAS, CD36, Gpam, and SREBP-1c. Furthermore, quercetin enhances cholesterol efflux from THP-1 macrophages by upregulating ABCA1 gene expression through activation of PPARγ and liver X receptor α (LXRα) pathways, representing a multifaceted approach to lipid management (220, 221). These mechanisms collectively position quercetin as a promising therapeutic agent for hyperlipidemia with potentially fewer adverse effects than conventional pharmacotherapies (222).

### 6.9 Gouty arthritis

Gouty arthritis (GA) is a non-infectious autoinflammatory disease triggered by the deposition of monosodium urate (MSU) crystals in joints and surrounding tissues due to persistently elevated serum uric acid levels (120). This condition is frequently associated with various comorbidities, including cardiovascular diseases, type 2 diabetes, and obesity. Current clinical management primarily relies on colchicine, non-steroidal anti-inflammatory drugs (NSAIDs), and corticosteroids, which, while providing rapid symptomatic relief, are often associated with significant gastrointestinal and renal toxicity, with colchicine being particularly notorious for its broad-spectrum toxic effects (121, 223).

Emerging research demonstrates that quercetin exerts significant therapeutic effects against MSU crystal-induced acute GA. In experimental models, quercetin markedly reduces joint swelling in GA rats, potentially through suppression of COX-2 protein expression (224). The compound competitively inhibits XOD activity, thereby blocking the catalytic conversion of xanthine and hypoxanthine to uric acid and effectively lowering serum urate levels. Furthermore, quercetin attenuates MSU crystal-induced ankle joint destruction in GA by downregulating key inflammatory mediators in the IL-17 pathway, including IL-6, IL-17A, and IL-17F. These multifaceted actions position quercetin as a promising therapeutic candidate for GA management with potentially fewer adverse effects than conventional therapies (225).

## 7 Clinical trials of quercetin in metabolic diseases

A randomized, double-blind, placebo-controlled crossover trial involving 37 healthy adults demonstrated that quercetin supplementation significantly reduced plasma levels of methylglyoxal, a compound associated with diabetes and its complications, suggesting potential benefits in diabetes management (226). Epidemiological data from a study of 10,054 Finnish citizens indicated that higher quercetin intake was associated with a lower risk of type 2 diabetes mellitus (T2DM) (227). Clinical evidence further revealed that quercetin significantly reduced serum low-density lipoprotein (LDL) levels in T2DM patients aged 30–60 years (228). A systematic review and meta-analysis concluded that quercetin intake at doses ≥500 mg/day for ≥8 weeks significantly lowered fasting blood glucose levels, highlighting the importance of dosage and duration in determining therapeutic efficacy (229).

In a 12-week randomized, double-blind, placebo-controlled trial assessing blood parameters in patients with MAFLD, quercetin supplementation significantly increased serum red blood cell (RBC) levels and reduced oxidative stress-induced RBC death, though it slightly decreased mean corpuscular volume, mean corpuscular hemoglobin, and ferritin levels (230–232). Clinical trials also demonstrated that quercetin reduced intrahepatic lipid content from 11.5 ± 6.4% to 9.6 ± 5.8% in MAFLD patients, correlating with weight loss and no adverse effects (233).

A 12-week randomized, double-blind, placebo-controlled trial involving 84 insulin-resistant women with PCOS showed that daily oral administration of 1000 mg quercetin improved IR and hormonal balance (234). Quercetin downregulated resistin gene expression in peripheral blood mononuclear cells and indirectly suppressed luteinizing hormone receptor and insulin receptor gene expression. Additionally, a randomized study of 24 patients with mild hyperlipidemia found that long-term quercetin intake reduced waist circumference, TC, LDL-C, and LDL-C/HDL-C ratio while enhancing total antioxidant capacity and delaying LDL oxidation, indicating favorable effects on lipid metabolism.

In a clinical trial, 22 healthy male volunteers with elevated plasma uric acid (but not hyperuricemia) received 500 mg/day of quercetin for 4 weeks. Their plasma uric acid levels decreased significantly by 26.5 μmol/L, without affecting fasting glucose, urinary uric acid excretion, or blood pressure, suggesting quercetin’s potential in preventing hyperuricemia. The clinical trial summary of quercetin is shown in **Table 2**. Current clinical trials investigating quercetin exhibit substantial methodological heterogeneity, which limits robust inference regarding its therapeutic efficacy. Systematic assessments indicate that although many studies adopt randomized, double-blind, placebo-controlled designs (Jadad score ≥3), sample sizes are generally small (median n≈37, range 22–90), resulting in limited statistical power to detect clinically meaningful endpoints and an increased risk of type II error (10). Additional methodological limitations include inadequate allocation concealment—only a minority of trials clearly reporting centralized randomization or coded assignment—suboptimal blinding procedures (e.g., open-label designs or mismatched placebos), and selective reporting bias arising from the absence of prospectively registered protocols and a tendency toward publication of positive findings. Participant selection bias is particularly prominent: most trials enroll healthy volunteers or individuals with single metabolic phenotypes (e.g., isolated hyperuricemia), while excluding patients with multimorbidity, hepatic or renal impairment, or concurrent polypharmacy, thereby limiting external validity (230). Short follow-up duration represents another major constraint. More than 80% of studies involve intervention periods of ≤12 weeks, whereas the chronic and progressive nature of metabolic diseases necessitates at least 6–12 months of observation to evaluate long-term safety, sustained tolerability, and clinically meaningful outcomes such as cardiovascular events or diabetic microvascular complications (233). Collectively, these sources of bias suggest that the current evidence base remains largely within the low-to-moderate certainty range and requires cautious interpretation under frameworks such as Grading of Recommendations Assessment, Development and Evaluation (GRADE).

**Table 2:** Clinical evidence of quercetin in metabolic diseases: human trials and patient outcomes.

Quercetin type	Dose	Disease type/population/duration	Outcome measures	Literature sources
Quercetin food additive	24.2 ± 26.7 mg/day	T2DM; 10,054 subjects; 1 year	Higher quercetin intake was associated with a reduced incidence of type 2 diabetes mellitus and related cardiovascular diseases.	(227),
Quercetin capsules	500 mg twice daily	MAFLD; 90 patients; 12 weeks	Erythrocyte quercetin concentrations increased significantly; peroxidative injury in erythrocytes was attenuated.	(231)
Quercetin capsules	500 mg once daily	MAFLD; 41 patients; 28 weeks	Intrahepatic lipid content decreased from 11.5 ± 6.4% to 9.6 ± 5.8%.	(230)
Quercetin capsules	500 mg twice daily	PCOS; 84 patients; 12 weeks	Insulin resistance and hormonal profiles improved; resistin gene expression in peripheral blood mononuclear cells was modulated; luteinizing hormone receptor and insulin receptor gene expression were indirectly suppressed.	(232)
Quercetin-rich onion juice	100 ml once daily	Hyperlipidemia; 24 subjects; 8 weeks	Waist circumference, total cholesterol, LDL-C, and the LDL-C/HDL-cholesterol ratio were reduced; total antioxidant capacity increased; the lag time for LDL oxidation was prolonged.	(230)
Quercetin dihydrate tablets	500 mg once daily	Hyperuricemia; 22 subjects; 4 weeks	Plasma uric acid concentration decreased significantly (by 26.5 μmol/L); fasting blood glucose, urinary uric acid excretion, and blood pressure remained unchanged.	(234)

Pharmacokinetic characteristics of quercetin further highlight the challenges associated with its clinical translation. Following oral administration, quercetin undergoes extensive phase II metabolism—including glucuronidation, sulfation, and methylation—such that the parent compound is rarely detectable in plasma. Circulating forms are predominantly conjugated metabolites, such as quercetin-3-glucuronide and quercetin-3′-sulfate, whose biological activities differ substantially from the aglycone (24). These metabolites also display limited tissue distribution and restricted penetration across physiological barriers, including the blood–brain barrier and adipose tissue—key target sites in metabolic and obesity-related complications. The pharmacokinetic profile is further complicated by enterohepatic recirculation and gut microbiota–mediated metabolism. Approximately 30–50% of conjugated metabolites are excreted into bile and subsequently hydrolyzed by microbial enzymes (e.g., β-glucuronidases) to regenerate the aglycone for reabsorption, prolonging the terminal half-life to roughly 16–20 hours but simultaneously introducing variability in exposure profiles. To address these limitations, formulation engineering strategies have shown promising improvements. For instance, phospholipid complexes (e.g., quercetin phytosome formulations) enhance micellar incorporation and can increase oral bioavailability by approximately twentyfold, achieving plasma concentrations exceeding ~1000 nM in healthy volunteers. Similarly, nanocrystal formulations and solid dispersions improve dissolution rate and saturation solubility, thereby overcoming the intrinsic low aqueous solubility of quercetin (~60 mg/L at 16 °C) (235). Nevertheless, clinical validation of these enhanced formulations remains at an early stage. Large-scale, long-term randomized controlled trials in patients with metabolic diseases—particularly those focusing on clinically meaningful outcomes—are still lacking, representing a critical evidence gap between mechanistic proof-of-concept and the establishment of quercetin as a standardized therapeutic intervention.

In summary, clinical evidence supports quercetin as a promising therapeutic candidate for metabolic disorders, demonstrating efficacy without significant adverse effects. However, clinical studies remain limited, and further research is needed to elucidate its precise mechanisms of action in humans.

## 8 Summary

Quercetin ameliorates metabolic diseases not through the isolated modulation of a single signaling pathway, but by orchestrating an integrated regulatory network centered on energy sensing, redox homeostasis, and inflammatory control. Within this framework, AMP-activated protein kinase (AMPK) functions as the central metabolic sensor and regulatory hub (47). Activation of AMPK by quercetin leads to phosphorylation and inhibition of acetyl-CoA carboxylase (ACC), thereby suppressing fatty acid synthesis while promoting fatty acid oxidation. Concurrently, AMPK activation enhances the deacetylase activity of SIRT1, forming an AMPK–SIRT1 positive feedback loop that suppresses proinflammatory signaling pathways, including NF-κB and the NLRP3 inflammasome, thereby attenuating chronic low-grade inflammation (85). In parallel, AMPK activation inhibits the mTOR pathway, relieving its negative regulation of autophagy and facilitating the clearance of damaged organelles and misfolded proteins—processes that are particularly relevant to the pathogenesis of MAFLD and atherosclerosis. Collectively, this AMPK-centered energy–inflammation–autophagy regulatory axis represents a unifying molecular basis underlying the pleiotropic metabolic benefits of quercetin.

Beyond this central axis, extensive crosstalk among quercetin-regulated pathways enables coordinated correction of multiple pathological components of metabolic dysfunction. In hepatic lipid metabolism, quercetin suppresses the mTOR/YY1 signaling axis, thereby relieving transcriptional repression of cholesterol 7α-hydroxylase (CYP7A1) and promoting the conversion of cholesterol to bile acids. This process operates in concert with bile acid signaling feedback mediated by FXR and TGR5, collectively maintaining cholesterol homeostasis. At the level of insulin signaling, quercetin activates the PI3K/Akt pathway to enhance GLUT4 membrane translocation and glucose uptake. This pathway exhibits functional synergy with AMPK signaling: AMPK enhances PI3K/Akt sensitivity, while Akt activation can reciprocally modulate AMPK activity, forming a network that supports metabolic flexibility. Importantly, quercetin also activates the Nrf2/ARE antioxidant response system, which counterbalances NF-κB–mediated inflammatory signaling. Activation of Nrf2 suppresses NF-κB nuclear translocation, whereas inhibition of NF-κB reduces inflammatory mediator–driven depletion of Nrf2 activity, thereby establishing a dynamic equilibrium between oxidative stress and inflammation (81). Through this multilayered integration of metabolic, oxidative, and inflammatory signaling pathways, quercetin interrupts the pathological cycle of “metabolic dysregulation–oxidative stress–chronic inflammation” characteristic of metabolic diseases, providing systemic rather than merely symptomatic therapeutic benefits.

**Figure 5:**
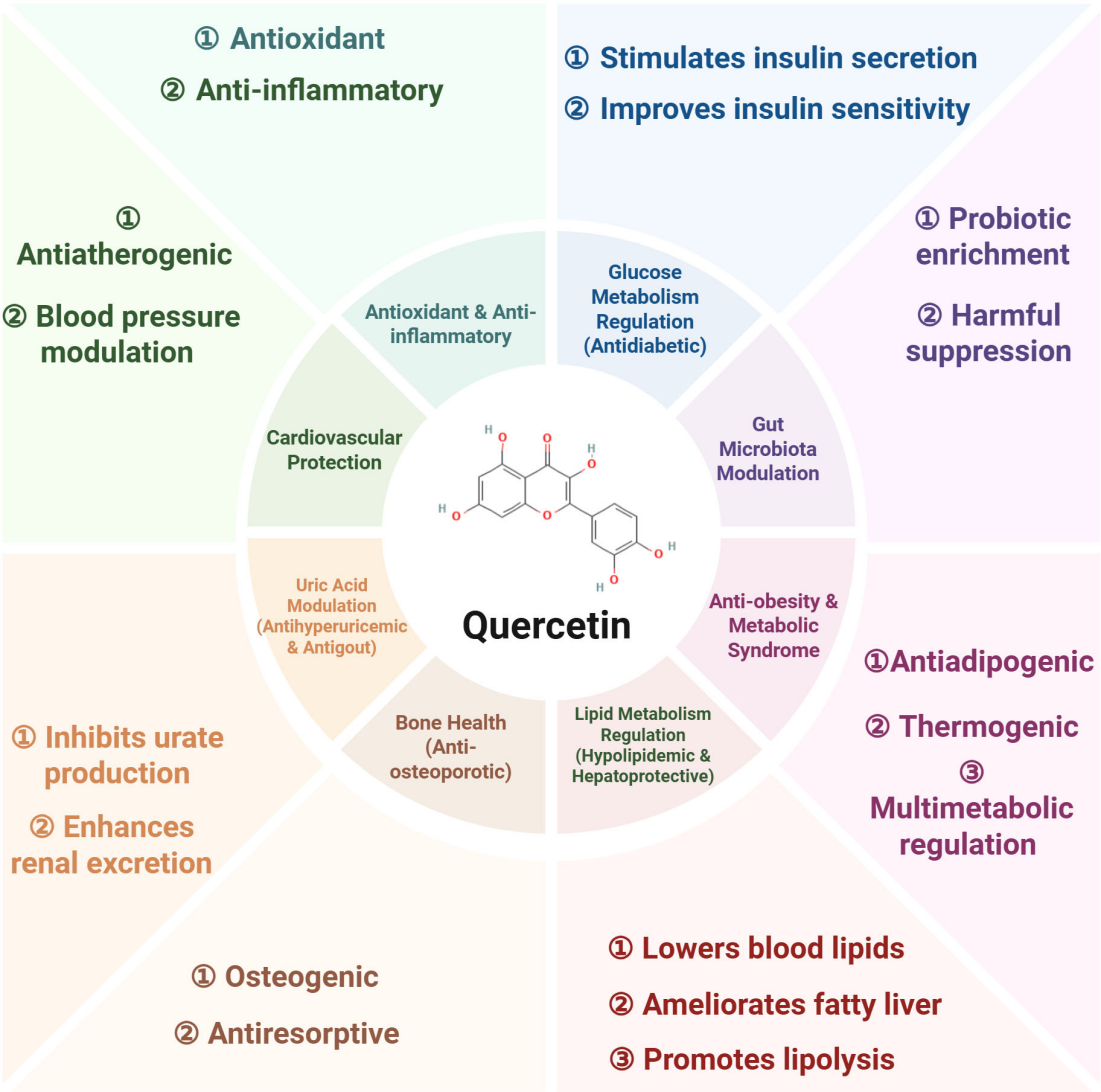
The main pharmacological effects of quercetin.

**Figure 6:**
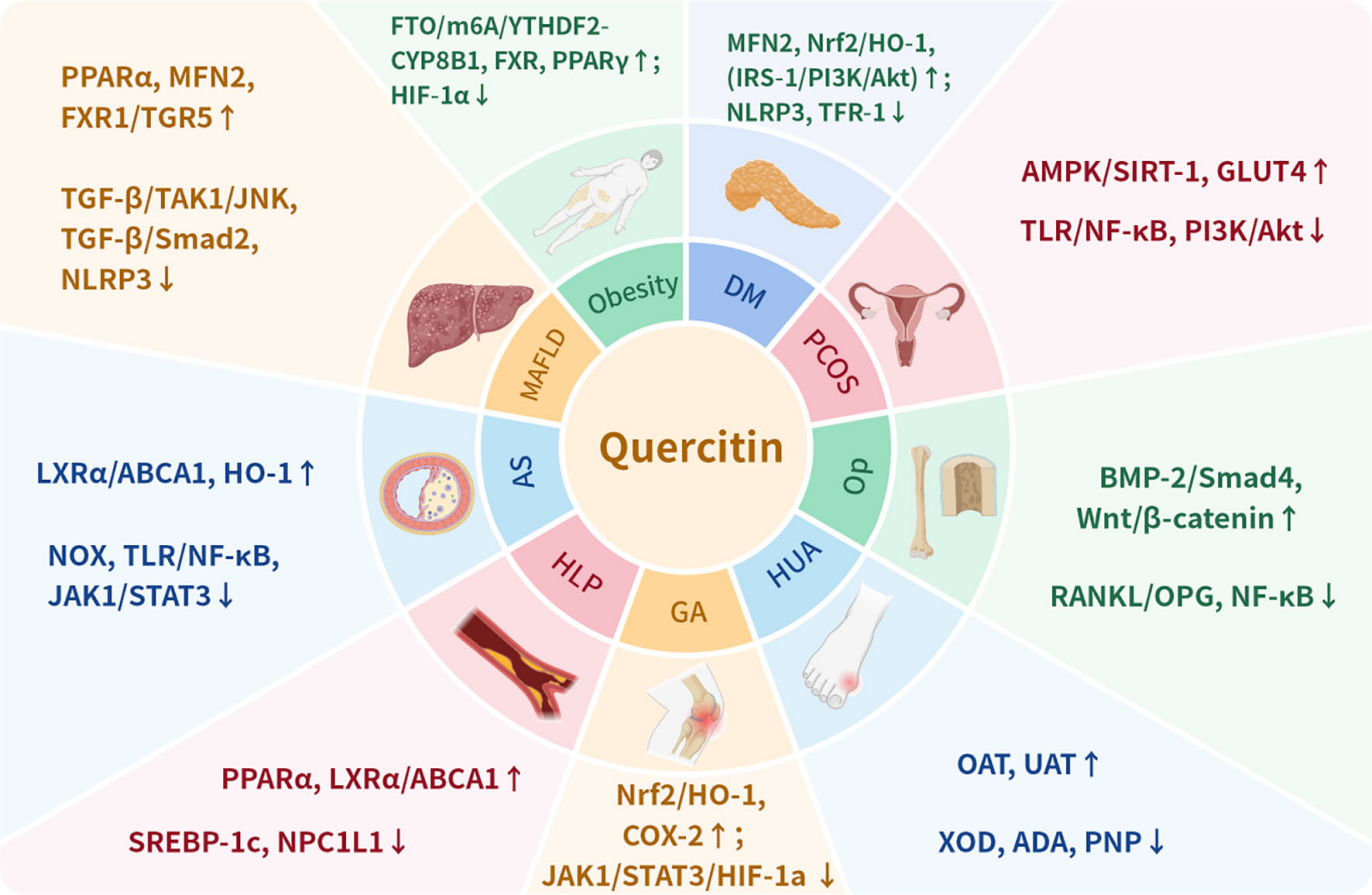
Mechanism of quercetin in treating metabolic diseases.

Although this review focuses on the multi-target regulatory mechanisms of quercetin (**Figures 5**, **6**), it should be noted that quercetin does not function as an isolated phytochemical. Other structurally related flavonoids—including quercitrin, kaempferol, rutin, apigenin, and catechins—also exert beneficial effects on metabolic diseases through shared or distinct molecular targets (236). This “class effect” highlights both the functional redundancy and structural diversity of flavonoids as a natural product reservoir, and provides a comparative framework for positioning quercetin in clinical translation. Compared with certain flavonoids whose clinical development is hindered by poor stability or extremely low bioavailability, quercetin exhibits a relative advantage in balancing pharmacological efficacy and drug-likeness; however, its absolute bioavailability remains substantially lower than that of most synthetic drugs. Therefore, the mechanistic network of quercetin summarized in this review can be regarded not only as a representative paradigm for flavonoid-mediated metabolic health improvement, but also as a conceptual basis for future head-to-head comparisons among different flavonoids and for the rational design of structurally optimized derivatives.

Although quercetin is generally well tolerated as a dietary flavonoid, a rigorous evaluation of its dose–response relationship, therapeutic window, and long-term safety remains essential for clinical translation. Preclinical studies indicate very low acute toxicity; for example, oral LD_50_ values in rodents exceed 10 g/kg, and 90-day feeding studies with dietary concentrations up to 5% have shown no significant adverse effects (9, 14). Pharmacological investigations also support its biological activity, as demonstrated by experimental evidence that quercetin attenuates ischemia–reperfusion–induced lung injury through modulation of oxidative stress and inflammatory signaling (237, 238). Nevertheless, high-dose exposure (generally >1000 mg/day) may paradoxically exert pro-oxidant effects under specific microenvironmental conditions—particularly in the presence of elevated transition metal ions—through redox cycling that generates semiquinone radicals and reactive oxygen species, potentially leading to DNA damage and mitochondrial dysfunction. Clinical trials using 500–1000 mg/day for 8–12 weeks have not reported serious adverse events, with only mild gastrointestinal discomfort observed in a small proportion of participants (<5%). However, robust safety data for prolonged use (>1 year) remain lacking. Potential renal risks should also be considered in susceptible populations, as high doses may inhibit renal organic anion transporters (OATs), thereby interfering with the excretion of uric acid and other metabolites and potentially aggravating accumulation-related injury in individuals with impaired renal function. Importantly, the safety evaluation framework for quercetin nanoformulations remains underdeveloped. Nanocarriers themselves may exhibit unique biodistribution, accumulation, or immunological effects, and potential long-term toxicities—such as lysosomal dysfunction or fibrosis—require independent systematic assessment rather than direct extrapolation from the toxicological profile of free quercetin.

Another critical translational issue concerns the potential for drug–drug interactions. Quercetin functions as a multi-target modulator of cytochrome P450 enzymes and drug transporters, raising the possibility of clinically relevant pharmacokinetic interactions. *In vitro* studies demonstrate that quercetin competitively inhibits CYP3A4, CYP2C9, and CYP1A2, thereby altering the metabolism of commonly prescribed drugs processed by these enzymes, including statins, warfarin, and sulfonylurea hypoglycemic agents, which may result in elevated systemic exposure and increased toxicity risk. Moreover, quercetin can inhibit intestinal P-glycoprotein (P-gp) and breast cancer resistance protein (BCRP), potentially enhancing the bioavailability of substrate drugs such as digoxin or methotrexate and altering their pharmacokinetic profiles. In the context of polypharmacy frequently observed in metabolic disease management, these interactions may have complex consequences. For instance, CYP3A4 inhibition by quercetin could enhance the lipid-lowering efficacy of atorvastatin but simultaneously increase the risk of myopathy or hepatotoxicity. Conversely, inhibition of hepatic uptake transporter OATP1B1 may reduce statin entry into hepatocytes and attenuate therapeutic efficacy (229). Similarly, although quercetin may exhibit synergistic glucose-lowering effects when combined with antidiabetic medications, such interactions could also increase the risk of hypoglycemia, particularly in elderly patients or those with renal impairment. These considerations highlight the importance of clearly distinguishing between quercetin’s roles as a dietary supplement and as a therapeutic agent, and underscore the need for pharmacogenomics-informed guidance for individualized use.

Long-term exposure risks and safety evaluation in special populations remain major gaps in the translational research landscape. Epidemiological cohort studies suggest that extremely high flavonoid intake (exceeding approximately 500 mg/day quercetin equivalents) may be associated with controversial links to certain hormone-sensitive malignancies, including thyroid cancer and estrogen receptor–positive breast cancer. Mechanistically, these associations may relate to the partial agonistic activity of quercetin toward estrogen receptors (ERα/ERβ) and its inhibitory effects on thyroid peroxidase (97). In pregnant or lactating women, quercetin can cross both the placental and blood–milk barriers, yet evidence regarding its effects on fetal development or long-term metabolic programming in offspring remains insufficient; therefore, high-dose supplementation is generally discouraged during these periods. For pediatric and adolescent populations with metabolic disorders—such as early-onset type 2 diabetes or adolescent metabolic dysfunction–associated fatty liver disease—clinical evidence regarding the efficacy and safety of quercetin interventions is almost entirely lacking, and extrapolation from adult studies remains uncertain given the unique metabolic demands of growth and development (10). From a regulatory science perspective, quercetin nanoformulations represent novel drug delivery systems that require dedicated evaluation frameworks. Key issues include manufacturing quality control (e.g., batch-to-batch consistency and long-term stability), nonclinical safety assessment (e.g., immunogenicity and biodegradability), and appropriate clinical development pathways—specifically whether independent phase I–III trials are required or whether existing data on free quercetin can partially inform evaluation. Future research should therefore establish integrated safety assessment frameworks spanning *in vitro*, *in vivo*, and clinical levels; develop physiologically based pharmacokinetic (PBPK) modeling tools to predict potential drug interactions; and promote internationally harmonized standards for product quality and clinical trial design. Such efforts will be critical for enabling the safe translation of quercetin-based interventions from experimental research to evidence-based medical practice (239).

In conclusion, quercetin represents a cost-effective natural compound with excellent potential for treating metabolic diseases. Structural modifications, particularly nanoformulations, could significantly enhance its stability, oral bioavailability, therapeutic efficacy, and duration of action. Future basic research should focus on elucidating the complete signaling networks underlying quercetin’s anti-metabolic disease effects, with thorough *in vitro* and *in vivo* validation. Furthermore, well-designed, large-scale, high-quality, multicenter clinical trials are needed to systematically evaluate the safety, toxicological profiles, and clinical efficacy of quercetin and its nanoformulations compared to conventional therapies in patients with metabolic disorders.
